# Mass Spectrometry Insight for Assessing the Destiny of Plastics in Seawater

**DOI:** 10.3390/polym15061523

**Published:** 2023-03-19

**Authors:** Olga V. Kuznetsova, Sergey N. Shtykov, Andrei R. Timerbaev

**Affiliations:** 1 Vernadsky Institute of Geochemistry and Analytical Chemistry, 119991 Moscow, Russia; 2 Institute of Chemistry, Saratov State University, 410012 Saratov, Russia; 3 Institute of Inorganic Chemistry, University of Vienna, 1090 Vienna, Austria

**Keywords:** plastic materials, seawater, IRMS, ICP-MS, fragmentation, degradation

## Abstract

Plastic pollution has become an increasingly serious environmental issue that requires using reliable analytical tools to unravel the transformations of primary plastics exposed to the marine environment. Here, we evaluated the performance of the isotope ratio mass spectrometry (IRMS) technique for identifying the origin of polymer material contaminating seawater and monitoring the compositional alterations due to its chemical degradation. Of twenty-six plastic specimens available as consumer products or collected from the Mediterranean Sea, five plastics were shown to originate from biobased polymeric materials. Natural abundance carbon and hydrogen isotope measurements revealed that biopolymers incline to substantial chemical transformation upon a prolonged exposure to seawater and sunlight irradiation. To assess the seawater-mediated aging that leads to the release of micro/nano fragments from plastic products, we propose to use microfiltration. Using this non-destructive separation technique as a front end to IRMS, the fragmentation of plastics (at the level of up to 0.5% of the total mass for plant-derived polymers) was recorded after a 3-month exposure and the rate and extent of disintegration were found to be substantially different for the different classes of polymers. Another potential impact of plastics on the environment is that toxic metals are adsorbed on their surface from the seashore water. We addressed this issue by using inductively coupled mass spectrometry after nitric acid leaching and found that several metals occur in the range of 0.1–90 µg per g on naturally aged plastics and accumulate at even higher levels (up to 10 mg g^−1^) on pristine plastics laboratory-aged in contaminated seawater. This study measured the degradation degree of different polymer types in seawater, filling in the gaps in our knowledge about plastic pollution and providing a useful methodology and important reference data for future research.

## 1. Introduction

Concerns about environmental plastic pollution are constantly growing with increasing apprehension that the mismanagement of plastic waste throughout the world poses another global threat to humankind [1,2]. While the actual extent of environmental occurrence and potential impacts of plastic debris remain unclear [3], it is evident that the bioavailability and toxicity of smaller particles are much greater compared with those of larger fragments [4,5]. Small sizes can be the result of degradation or fragmentation of larger polymeric materials upon their disposal into and exposure to the marine environment, where they readily undergo various weathering or aging processes, including sunlight irradiation, wave and wind stress, mechanical abrasion, etc. Another source of microplastic contamination is the production and disposal of manufactured (primary) microplastics. It is also important to comprehend that the hazard potential of microplastics is not only because of their small size, increased uptake and reactivity [6], but also arises due to various plastic additives, such as persistent organic pollutants [7], catalytic remnants, polymerization solvents, etc. [8], or adsorbed toxic metals, which can leach into seawater and be consumed by marine organisms [9,10]. 

There is a continuum of societal response measures that can be taken against environmental hazards associated with nano- and microscale plastic pollution. Already implemented in many countries are product bans for small plastics, such as microbeads used in cosmetic products, as well as larger single-use plastic items (e.g., bags and straws) that can degrade into microplastics. In this context, a further regulation in the works implies the inclusion of nanoplastics in existing regulatory frameworks [11]. Perhaps more effective would be at least partial replacement of fossil-based plastics with those that completely decompose/degrade in natural ecosystems. Biopolymers are examples of such materials whose production has continued to grow exponentially in recent years [12,13]. Ultimately, to adequately address the issue of protecting the environment and human health, governmental and regulatory bodies should rely on reliable information on the occurrence of microplastics in marine environments. However, the implementation of monitoring programs is so far restricted by the lack of standardized methods and best-practice guidelines for the identification, quantification and characterization of micro- and nanoplastics. The problem of generating consistent analytical results is aggravated by the unique behavior of environmental microplastics that occur as non-homogeneously distributed particulates and fibers composed of various polymer types and are diverse in terms of their size, shape, surface properties, etc. [14]. 

From the large body of review literature [15,16,17,18,19,20,21], it appears that GC-MS coupled with pyrolysis (for plastic thermal degradation) holds promise for being advanced to standard methodology status. This is no great surprise, as the analytical measurements of microplastics basically vary little from the general methodology used for analyzing polymers, where GC-MS represents the most recognized technique. Notably, the same technique dominates the analysis of organic plastic additives originating from microplastics [20,22] and can also be used to assess their contamination with organic pollutants [23]. However, the GC-MS method is not free from limitations [24]. Most importantly, it cannot differentiate petroleum-based and biopolymeric materials to reveal their origin and fate in real environments such as seawater. In addition, most of the toxic metals that tend to be chemically adsorbed on microplastics [20] fall beyond the scope of GC-MS. 

To tackle these insufficiently studied issues, we employed here, as alternative mass-spectrometry-based tools, isotope ratio MS (IRMS) and inductively coupled plasma MS (ICP-MS). It was supposed that the IRs of light elements (such as carbon and hydrogen) would be different for plastic materials of diverse origins and, possibly, locally enriched or depleted upon fragmentation under the influence of various environmental factors. In this regard, it is worth noting the ability of IRMS to detect minute isotopic changes (0.001%) at nanomolar analyte concentrations and the requirement of very small samples. In its turn, ICP-MS has a proven record in marine metal analysis, including recent advances in monitoring challenging nanometal contaminants [25,26]. Essential (and discussed below) is another (and apparently a single) contribution focused on unveiling the seawater transformations of plastic polymers using IRMS [27]. 

## 2. Materials and Methods

### 2.1. Plastic Samples

Sample designation and description is given in Supplementary Materials, Table S1. Pristine plastic items, both petroleum-derived and biobased polymers (P1–P9 and B1–B6, respectively), were purchased from local department stores and not used for other purposes. All naturally aged samples (aged for an undefined period of time; N1–N11) were taken in July 2022 from the Mediterranean Sea (the beach of Viareggio, Viareggio, Italy) and stored separately for chemical characterization.

### 2.2. Sample Treatment

Plastics aged under laboratory conditions were prepared by placing the polymer materials under real seawater and gentle mechanical stirring for up to 3 months (room temperature, natural ambient light). The coastal seawater used in these and other experiments was collected, treated and analyzed as described elsewhere [28]. After fixed periods of time (15, 30, 45, 60, 75 and 90 days), each sample was removed, washed with ultrapure water and dried at a temperature of 40 °C. A small part (≤4 mm longwise and weighing less than 3 mg) was cut off from the sample by a scalpel for subsequent IRMS analysis (see below). Then, the rest of the sample was subjected to further seawater treatment.

For additional evaluation of marine degradation, an aliquot of the water layer over each sample was filtrated through a 10 kDa cut-off filter (Sigma 1-14k, Sigma Laborzentrifugen, Osterode am Harz, Germany) for 15 min at 10,000 rcf to separate the nano/microsized fraction of the fragmented material. The isolated fragments were removed from the filter unit by reverse filtration (after adding ultrapure water), dried under nitrogen gas stream in an evaporator–condenser (Supelco, Bellefonte, PA, USA) and subjected to analysis. High-purity nitrogen (>99.99%; from NIIKM, Moscow, Russia) was used for drying. 

For metal analysis, pristine plastic samples exposed to seawater were taken out after 6 h or 3 months, rinsed with ultrapure water, dried and placed into 30% (*v*/*v*) nitric acid solution. After 2 h of sonication, each sample was removed and the remaining solution diluted 10 times. The aged samples of N-series were analyzed similarly but without seawater treatment.

### 2.3. IR Measurements

Each plastic sample was divided into individual fragments (0.2–0.4 mg), weighted prior to analysis on microanalytical balances (Balance XPR56/A, Mettler Toledo, Greifensee, Switzerland) and placed into tin or silver capsules. The capsules were gently crimped, closed and placed in the autosampler. The carbon stable isotope composition was determined using a Delta Plus XP mass spectrometer interfaced with a Flash 2000 (both from Thermo Fisher Scientific, Bremen, Germany) at oxidation and reduction furnace temperatures of 1200 and 650 °C, respectively. The measurements of ^2^H/^1^H ratios (δD) were performed with a system consisting of a Thermo Combustion unit coupled with a Delta Plus XP via a ConFlo III-Interface (both from Thermo Fisher Scientific) at the following operative conditions: reactor temperature, 1350 °C; GC column temperature, 90 °C; helium flowrate, 90 L min^−1^. The accuracy of the isotopic data was evaluated by analyzing the certified reference material IAEA-CH-7 (a PE foil with δ^13^C = −31.8 ± 0.2‰ and δD = −99.2‰) obtained from International Atomic Energy Agency, Vienna, Austria. High-purity helium and hydrogen gases (>99.9999%; from NIIKM) and CO_2_ (≥99.999%; Voessen, Moscow, Russia) were used as carrier gas or working standard gas, respectively. The same stable isotope measurements were performed following time-dependent seawater treatment of pristine samples after rinsing with ultrapure water and drying. 

### 2.4. Metal Analysis 

The metal content of the plastic samples was confirmed by high-resolution ICP-MS using an Element 2 instrument (Thermo Fisher Scientific, Waltham, MA, USA), operating in low- (*R* = 300) or medium-resolution setting (*R* = 4000) and the following instrumental settings: plasma gas flow, 14 L min^−1^; auxiliary gas flow, 0.9 L min^−1^; nebulizer gas flow, 0.9 L min^−1^; analyzed sample flow, 0.8 L min^−1^; RF power, 1250 W; dwell time, 20 ms. The internal standard ^115^In was analyzed to correct for non-spectral interferences during analysis.

### 2.5. Data Analysis

The analyses of all samples were performed in triplicates, with 5–10 (IRMS) and 3 (ICP-MS) parallel measurements, and expressed as the mean ± standard deviation. The latter was typically less than 0.2 and 1.5‰ for δ^13^C and δD, respectively. Statistical analysis of differences in metal concentrations amongst different contaminated and pristine samples was conducted by one-way analysis of variance using standard ANOVA program. 

## 3. Results and Discussion

### 3.1. Optimization of IRMS Measurements 

As a prerequisite for reliable isotopic assaying, it was necessary to understand how efficient the conversion of polymers into simple gases (CO_2_ and H_2_) is with sample masses chosen for analysis. If the mass turned out to be excessive, incomplete oxidation or reduction might take place, thus impairing the accuracy of the results. From the data of Table S2, it is evident that with the masses of IAEA-CH-7 reference material ranging from 0.21 to 5.15 mg, there is no statistically significant variation in the values of δ^13^C and δD, which amount to −31.76 ± 0.21‰ and −99.21 ± 0.90‰, respectively (cf. the respective certified values of −31.8 and −99.2). This confirms the quantitative conversion of PE into carbon dioxide and molecular hydrogen and provides a rationale for the mass range employed in this study (see Section 2.2). 

**Figure 1:**
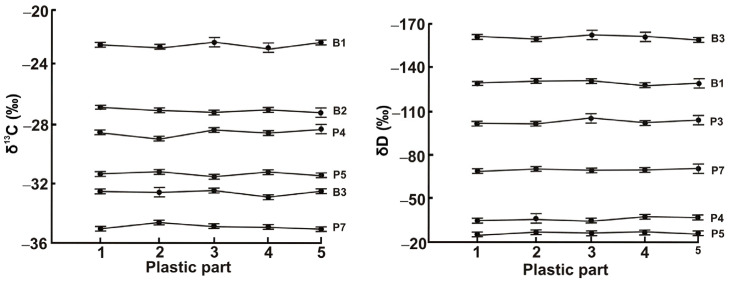
Carbon and hydrogen IRs for different parts of a representative selection of polymer samples. Error bars represent standard deviations.

Compositional uniformity is a key factor in the successful analysis of solid samples. To confirm that the polymer materials of interest met this requirement, five subsamples taken from different parts of each plastic material were independently analyzed. As can be seen from the data shown in Figure 1, the measured isotopic composition is characterized by slight differences in the δ^13^C and δD values but the standard deviations remain lower than 0.2 and 1.4%, respectively. Such variation thresholds witness the consistent isotopic composition of the studied polymers. 

### 3.2. Identification of Plastic Material

Yet before exploring the fragmentation/degradation processes, it was essential to identify the origin of the plastic samples collected from the sea as well as to validate the accuracy of the IRMS method with regard to the polymer materials with known compositions. Another point of interest was acquiring the IR data for plant-derived plastics, as systematic studies on such polymeric materials are lacking in the literature. Table 1 gives a comparison of δ^13^C values, measured for the samples of natural plastic debris, with the published data for common polymers. It seems that the majority of natural plastics can be ascribed to PP or PE, while only a few samples are attributed to other polymers, probably PTFE and PVC. However, in view of fairly small differences in the δ^13^C values of typical polymers (e.g., PE and PP), our assignment has only a tentative character. Another interesting observation is that regardless of the polymer family, most aged materials exhibit less negative values of δ^13^C as compared with pristine plastics, as is discussed below. 

The carbon isotopic composition of proprietary fossil-derived plastics (see the P-series in Table S1) is in the most cases in good agreement with the available literature data [27,29]. The only exception was observed for the PTFE sample (P9), whose δ^13^C (−34.57 ± 1.13‰) is less negative than the value reported by Berto et al. (−40.7 ± 1.17‰) [27]. This is probably due to a different production cycle or specific additives to the sample (cooking mat). In this regard, it should be noted that the isotopic composition of carbon can notably vary for the same polymer produced in different countries (e.g., up to 2% for PE [29]). The least negative δ^13^C value was obtained for the polylactide-based plastic (B5; −15.43 ± 0.32‰), which is consistent with its plant origin, most likely a corn starch derivate characterized by δ^13^C ranging from −8 to −20‰ [29]. For the B3 sample (biobased low-density PE), the carbon isotopic composition (−30.51 ± 0.42%) agrees well with the reported value for this type of polymer (−30.19 ± 1.56‰) [27]. Other B-type polymers under examination display isotopic shifts toward less negative values of δ^13^C (relative to pure PP) due to their production based on corn starch and agglomerated PP.

The hydrogen isotope analysis produces much wider data scattering and for this reason is less useful for the identification and discrimination of polymer materials. For instance, Jones et al. [30] examined the 26 samples of PE and found that the δD values vary in the range from −109.61 to −6.03‰. Our results also show significant hydrogen isotope variability, viz., from −121.58 to −46.91‰ in the case of PE.

### 3.3. Seawater-Induced Degradation

**Figure 2:**
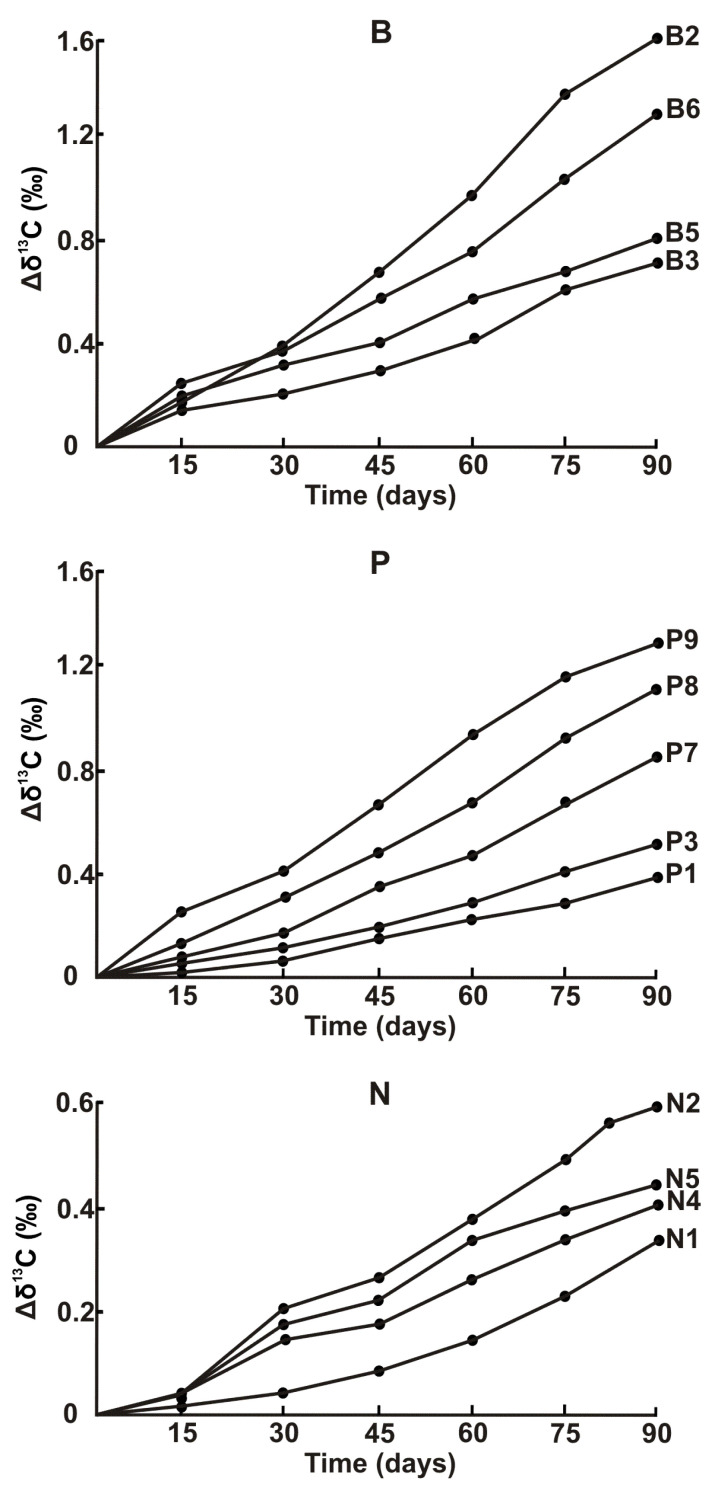
Degradation of polymer plastics reflected by the time-dependent variations in δ^13^C. Indices B, P and N correspond to the respective polymer classes.

As was expected, the results of IRMS analysis of polymers exposed to a simulated marine environment reveal definite isotopic alterations related to degradation, possibly promoted by the swelling phenomenon [31]. Shown in Figure 2 are changes in Δδ^13^C observed over three months of experimental seawater exposure (similar dependences for ΔδD are depicted in Figure S1). The shift of δ^13^C toward gradually less negative values, i.e., a depletion of ^13^C (or an enrichment of ^12^C), is evident with increased exposure time. It is interesting to note that alterations in isotopic composition are variable across polymer families, with the greatest changes occurring for biodegradable plastics (average Δδ^13^C and ΔδD are 1.18 and 15.1‰, respectively), followed by ‘non-bio’ (0.69 and 12.6‰) and then naturally aged (0.47 and 11.5‰) materials. For the latter type of plastics, the signs of certain aging due to a (lengthy) period residing in seawater may account for a modest inclination to further compositional changes. As a matter of fact, there are several mechanisms underlying seawater plastic degradation (photooxidation, biodegradation by microorganisms, chemical–mechanical degradation, hydrolysis, etc. [32,33,34]), and only some of these can be simulated in a laboratory setting. Furthermore, while the IRMS measurements performed in this study and by other researchers [27] provide a background for highlighting a degradation process, no exact degradation pathway involved in shifting isotopic values can in effect be differentiated. Nonetheless, our results give a clue to the degradation behavior of different polymer materials and an estimate of the time of their existence in and eventual removal from the sea, which are still poorly understood. 

In this context, it was essential to track the fragmentation process in time. For biobased polymers as well as for two samples of aged plastic (N7 and N10), the appearance of fibrous-like fragments in the overlaying seawater was evident after 40 days of observation and the amount of released debris increased by the end of the examination. On the other hand, no generation of (micro)plastic fibers or particles was distinguished for other aged plastics. The decomposition rate of durable pristine polymers was likewise too slow to record debris release under simulated seawater conditions. After isolation by combined microfiltration/reverse filtration (an approach that proved effective for the separation of nanoparticles from seawater [28]), the disintegrated material was subject to IRMS analysis. The data of Table 2 reveal that the fragmentation of biodegradable polymers is associated with less negative values of δ^13^C (by 0.7–1.8%) and δ^13^D (by 10–22%; not shown in the table for the sake of conciseness). For instance, the isolated fragments of corn-based plastics (B1, B2 and B6) by their isotopic composition approach starch, which is likely due to abiotic or biotic degradation. To assess the extent of in-force fragmentation, direct mass measurements were carried out for bioplastic samples before and after the 3-month exposure to seawater. As can be seen from Table 2, the greater the mass loss, the larger the shift of IR data is.

### 3.4. Metal Contamination

Plastic debris, before transforming into the micro/nanoplastic state, affects the transfer of metals in marine ecosystems by adsorbing a variety of trace metals [35]. The first essential step—before the identification and determination of possible metal contaminants—was to devise a sample-treatment strategy suitable to isolate them from plastic material. We applied here nitric acid leaching following a careful optimization of acid concentration. As a matter of fact, its increasing would ensure the quantitative desorption of metals but inevitably require higher dilution of the leachate, posing the risk that an actual concentration would fall beyond the limit of quantification. From the data of Table S3, it appears that there is no need to use HNO_3_ higher than 30% (*v*/*v*) to attain maximum metal recoveries. 

To gain insight into the extent of metal contamination, we devised two experimental series with the samples of contaminated and pristine plastic material. While the former, naturally aged polymer samples were analyzed directly, without additional seawater treatment, the pristine plastics were exposed to seawater, which was notably collected in close proximity to a highly urbanized city with heavy industries [28]. It is worthwhile to note that since the N-series samples were aged for an unidentified period of time (and taken from a beach area), the acquired data only have a comparative character. Nevertheless, as shown in Table 3, some of the samples were found to display an exceptionally high level of contamination, particularly with toxic metals such as Cu and Pb. This is especially true for sample N10 (a colored food package), in which extreme values were measured for six metals. 

Exploring the accumulation of metals on plastic materials as a function of their residence time in contaminated seawater revealed several general tendencies. First and perhaps most discouraging is that some plastic materials already contain substantial amounts of metals before encountering the seawater setting (zero-time data in Table 4). This is the case for sample B2 (make-up sponge), whose starch-based material is contaminated with a wide range of metals, and to a lesser degree, samples B1 and B6. Next, all the materials under scrutiny tend to accumulate metals upon prolonged laboratory aging, with plant-derived plastic polymers exhibiting greater metal affinity (mainly those that incline to greater fragmentation; see, e.g., the data for B2). In several instances, this leads to remarkably high metal contents, i.e., 10 mg g^−1^ and higher. Such acute concentrations seemingly originate on account of contaminated seawater, and in the case of open seawater might be substantially lower than presently determined. Another important observation is that different metals incline to a varying degree of accumulation. It is also worth noting that minor seawater metals, while steadily transferring into an immobilized state over time, do not, with a few exceptions, exceed the levels of naturally aged plastics as high as more abundant Cu, Fe, V and Zn. 

## 4. Conclusions

IRMS proved useful as a ‘one-stone-three-birds’ tool for the characterization of plastic polymers in a seawater environment. The method was shown capable of (i) acquiring reliable IR data that help with the identification of polymer debris contaminating seawater, (ii) differentiating between petroleum- and plant-derived polymers, and (iii) monitoring their compositional alterations due to chemical degradation in seawater. The main novelty element of the present study with regard to published IRMS-based research [27,29,36] is the direct analysis of the polymer material disintegrated upon exposure to seawater. Nonetheless, the proposed approach that integrates the IRMS method with single-membrane microfiltration is in principle incapable of discerning nano-, micro- or larger-size fragments. To address this unresolved issue, our upcoming research will implement an experimental design based on cascade filtration (with filters of different pore sizes), which has already found a niche in the analysis of microplastics [37]. Another research avenue to be pursued before long is research toward unveiling the structural changes in plastics during degradation as well as the structure of disintegrated fragments. With this goal in mind, we will rely on using a selection of complementary spectroscopic techniques such as Fourier transform IR [15], laser direct IR [38] and NMR [8]. 

## Figures and Tables

**Table 1:** Natural plastic polymers and their carbon isotopic signatures.

Sample	δ^13^C (‰)	Assigned Composition ^a^	Respective δ^13^C (‰)
N1	−26.91 ± 0.51	PP	−28.0 ± 1.5 [27]
N2	−28.27 ± 0.53	PE or PP	−28.0 ± 1.5 [27]
N3	−27.58 ± 0.62	PP	−28.0 ± 1.5 [27]
N4	−28.48 ± 0.50	PVC	−28.5 ± 1.5 [29]
N5	−28.80 ± 0.91	PE	−29.3 ± 1.1 [27]
N6	−28.89 ± 0.77	PE	−29.3 ± 1.1 [27]
N7	−38.79 ± 1.27	PTFE	−40.2 ± 1.2 [29]
N8	−27.51 ± 0.56	PP	−28.0 ± 1.5 [27]
N9	−27.06 ± 0.77	PP	−28.0 ± 1.5 [27]
N10	−29.07 ± 1.11	PE	−29.3 ± 1.1 [27]
N11	−26.96 ± 1.14	PP	−28.0 ± 1.5 [27]

^a^ PP = polypropylene; PVC = polyvinylchloride; PTFE = polytetrafluoroethylene.

**Table 2:** Characteristics of the fragmentation of plant-derived plastic polymers in seawater.

Sample	δ^13^C (‰)		Mass (g)		Mass Loss (%)
	Initial	After 3 Months	Initial	After 3 Months	
B1	−22.42 ± 0.42	−21.04 ± 0.59	0.39343	0.39187	0.40
B2	−26.07 ± 0.71	−24.44 ± 1.13	0.41561	0.41322	0.58
B3	−30.51 ± 0.44	−29.78 ± 0.41	0.68275	0.68251	0.04
B4	−28.92 ± 0.36	−27.61 ± 0.77	0.23454	0.23411	0.18
B5	−15.43 ± 0.47	−14.61 ± 0.53	1.23363	1.23140	0.18
B6	−25.62 ± 1.01	−24.38 ± 0.61	0.46944	0.46825	0.25

**Table 3:** Metal concentrations in acidic rinses of contaminated plastic material (µg g^−1^) ^a^.

Sample	Cd	Co	Cu	Cr	Fe	Mn	Mo	Ni	Pb	Ti	V	Zn
N1	0.10 ± 0.01	5.4 ± 0.2	22 ± 1	38 ± 3	91 ± 8	6.0 ± 0.5	0.80 ± 0.03	9.7 ± 0.5	3.6 ± 0.3	0.6 ± 0.03	0.30 ± 0.01	14 ± 1
N2	0.10 ± 0.01	3.3 ± 0.2	14 ± 1.	5.2 ± 0.5	77 ± 6	6.2 ± 0.5	0.30 ± 0.02	5.5 ± 03	1.4 ± 0.1	0.8 ± 0.04	0.20 ± 0.01	18 ± 1
N3	0.02 ± 0.001	2.9 ± 0.2	11 ± 1	4.6 ± 0.4	89 ± 8	5.1 ± 0.4	0.30 ± 0.01	3.7 ± 0.2	1.6 ± 0.2	0.3 ± 0.01	0.10 ± 0.01	14 ± 1
N4	0.70 ± 0.02	1.3 ± 0.1	6.8 ± 0.3	5.3 ± 0.5	92 ± 9	4.9 ± 0.4	0.20 ± 0.02	3.5 ± 0.2	1.4 ± 0.1	0.25 ± 0.03	0.10 ± 0.01	15 ± 1
N5	0.03 ± 0.002	0.20 ± 0.01	2.4 ± 0.2	**44 ± 3**	79 ± 8	8.1 ± 0.6	0.30 ± 0.02	5.5 ± 0.4	2.4 ± 0.1	**66 ± 5**	0.20 ± 0.01	17 ± 2
N6	0.02 ± 0.001	1.1 ± 0.05	5.5 ± 0.4	4.9 ± 0.5	90 ± 6	6.2 ± 0.5	0.20 ± 0.01	4.5 ± 0.3	0.50 ± 0.03	0.6 ± 0.04	0.20 ± 0.01	15 ± 1
N7	0.10 ± 0.005	1.3 ± 0.1	4.6 ± 0.3	5.1 ± 0.4	102 ± 9	**56 ± 5**	1.6 ± 0.1	5.2 ± 0.4	5.9 ± 0.4	3.2 ± 0.2	0.50 ± 0.04	16 ± 1
N8	0.10 ± 0.003	1.1 ± 0.05	6.2 ± 0.4	11 ± 1	98 ± 8	2.4 ± 0.2	0.40 ± 0.03	6.6 ± 0.5	0.30 ± 0.02	0.2 ± 0.01	0.10 ± 0.01	19 ± 1
N9	0.10 ± 0.004	2.2 ± 0.2	**47 ± 4**	6.1 ± 0.4	105 ± 9	9.1 ± 0.8	0.40 ± 0.02	**88 ± 8**	1.2 ± 0.1	8.2 ± 0.6	0.90 ± 0.02	22 ± 1
N10	**3.2 ± 0.3**	2.6 ± 0.1	**77 ± 6**	13 ± 1	126 ± 8	**55 ± 4**	1.3 ± 0.05	**27 ± 2**	**68 ± 5**	10 ± 1	**4.3 ± 0.3**	18 ± 1
N11	0.20 ± 0.01	5.3 ± 0.2	**90 ± 8**	8.0 ± 0.6	99 ± 8	8.2 ± 0.7	0.80 ± 0.03	13 ± 1	**8.7 ± 0.7**	1.2 ± 0.1	0.30 ± 0.02	24 ± 1

^a^ The outliers are given in bold face (here and below).

**Table 4:** Time-dependent contamination of pristine plastic material with seawater metals (µg g^−1^).

Sample	Time ^a^	Cd	Co	Cu	Cr	Fe	Mn	Mo	Ni	Pb	Ti	V	Zn
B1	0	0.03 ± 0.001	0.10 ± 0.01	1.7 ± 0.1	2.0 ± 0.1	11 ± 0.2	1.0 ± 0.02	0.10 ± 0.01	1.3 ± 0.1	0.60 ± 0.03	0.20 ± 0.01	0.10 ± 0.01	3.1 ± 0.3
	6 h	0.40 ± 0.03	1.0 ± 0.1	27 ± 4	22 ± 0.5	**2860 ± 20**	**397 ± 25**	1.3 ± 0.03	12 ± 0.5	13 ± 0.1	**3300 ± 230**	7.6 ± 0.3	**93 ± 7**
	3 m	0.50 ± 0.03	5.1 ± 0.4	39 ± 3	26 ± 2	**3390 ± 240**	**510 ± 42**	3.4 ± 0.4	49 ± 4	28 ± 2	**4400 ± 270**	15 ± 1	**161 ± 13**
B2	0	**2.2 ± 0.2**	**3.3 ± 0.2**	**57 ± 4**	**30 ± 2**	**144 ± 9**	**59 ± 5**	0.30 ± 0.02	7.0 ± 0.1	**60 ± 5**	0.9 ± 0.1	0.4 ± 0.02	19 ± 2
	6 h	**25 ± 0.6**	**26 ± 3**	**841 ± 32**	**757 ± 9**	**8330 ± 2100**	**1800 ± 140**	1.7 ± 0.1	43 ± 21	**600 ± 27**	117 ± 3	13 ± 3	**696 ± 24**
	3 m	**28 ± 2**	**42 ± 3**	**1330 ± 110**	**1500 ± 110**	**8890 ± 770**	**2640 ± 200**	3.6 ± 3	78 ± 6	**624 ± 70**	157 ± 13	19 ± 2	**862 ± 66**
B3	0	0.03 ± 0.001	0.10 ± 0.01	4.7 ± 0.3	1.2 ± 0.05	14 ± 1	5.1 ± 0.2	0.10 ± 0.01	2.1 ± 0.2	1.4 ± 0.1	3.2 ± 0.3	0.10 ± 0.01	10 ± 0.5
	6 h	1.2 ± 0.1	6.1 ± 0.4	97 ± 2	13 ± 0.5	755 ± 41	112 ± 12	1.1 ± 0.1	27 ± 1	16 ± 0.2	83 ± 2	2.3 ± 0.1	14 ± 1
	3 3 m	1.3 ± 0.1	14 ± 1	**184 ± 14**	19 ± 1	814 ± 59	185 ± 14	4.6 ± 0.3	57 ± 5	40 ± 3	116 ± 9	3.8 ± 0.3	16 ± 1
B4	0	0.02 ± 0.001	0.20 ± 0.01	4.1 ± 0.4	1.1 ± 0.05	18 ± 2	3.0 ± 0.2	0.10 ± 0.01	4.6 ± 0.4	5.0 ± 0.3	1.1 ± 0.1	0.10 ± 0.01	1.0 ± 0.05
	6 h	1.2 ± 0.1	6.4 ± 0.2	**432 ± 6**	54 ± 4	**2720 ± 250**	56 ± 4	2.5 ± 0.1	61 ± 3	168 ± 7	13 ± 0.2	3.5 ± 0.6	2.5 ± 0.2
	3 m	1.3 ± 0.1	9.5 ± 0.8	**576 ± 47**	103 ± 9	**4320 ± 260**	150 ± 11	6.6 ± 0.5	94 ± 8	196 ± 17	21 ± 1	7.3 ± 0.5	11 ± 1
P1	0	0.01 ± 0.001	0.1 ± 0.01	3.1 ± 0.2	1.3 ± 0.1	22 ± 2	2.0 ± 0.2	0.01 ± 0.001	2.4 ± 0.1	6.0 ± 0.4	0.30 ± 0.02	0.10 ± 0.01	3.0 ± 0.2
	6 h	0.40 ± 0.05	0.9 ± 0.1	71 ± 1	21 ± 2	530 ± 61	25 ± 2	0.20 ± 002	45 ± 3	116 ± 2.3	5.1 ± 0.6	2.0 ± 0.2	**179 ± 2**
	3 m	0.50 ± 0.03	1.8 ± 0.2	117 ± 12	47 ± 4	772 ± 62	29 ± 2	0.30 ± 0.02	74 ± 5	139 ± 10	11 ± 1	3.3 ± 0.2	**190 ± 14**
P2	0	0.01 ± 0.001	0.01 ± 0.001	4.0 ± 0.3	0.30 ± 0.02	23 ± 2	2.0 ± 0.2	0.10 ± 0.01	3.4 ± 0.2	1.6 ± 0.1	0.20 ± 0.02	0.10 ± 0.01	6.0 ± 0.4
	6 h	0.30 ± 0.02	0.80 ± 0.10	49 ± 1	8.6 ± 0.5	295 ± 25	38 ± 3	2.0 ± 0.3	37 ± 2	42 ± 3	2.0 ± 0.1	1.7 ± 0.1	**188 ± 4**
	3 m	0.35 ± 0.03	1.2 ± 0.1	55 ± 4	9.7 ± 0.9	411 ± 37	50 ± 4	2.6 ± 0.2	44 ± 3	61 ± 5	2.7 ± 0.2	2.1 ± 0.1	**203 ± 16**
P3	0	0.01 ± 0.001	0.01 ± 0.001	2.0 ± 0.2	0.20 ± 0.02	13 ± 1	1.0 ± 0.1	0.02 ± 0.001	2.0 ± 0.2	3.0 ± 0.2	0.05 ± 0.003	0.05 ± 0.001	11 ± 0.4
	6 h	0.30 ± 0.02	0.70 ± 0.10	58 ± 2	8.3 ± 0.3	225 ± 19	36 ± 4	2.0 ± 0.1	41 ± 2	40 ± 1	2.5 ± 0.1	1.8 ± 0.1	**156 ± 4**
	3 m	0.35 ± 0.03	0.90 ± 0.04	66 ± 5	11 ± 1	330 ± 24	44 ± 3	2.6 ± 0.2	55 ± 4	67 ± 6	3.1 ± 0.2	2.2 ± 0.2	**199 ± 18**
P4	0	0.01 ± 0.001	0.20 ± 0.01	4.5 ± 0.3	2.3 ± 0.2	20 ± 2	1.4 ± 0.1	0.01 ± 0.001	1.4 ± 0.1	4.0 ± 0.3	0.10 ± 0.01	0.10 ± 0.01	5.0 ± 0.5
	6 h	0.30 ± 0.03	3.2 ± 0.4	58 ± 2	19 ± 1	231 ± 23	26 ± 3	1.3 ± 0.06	34 ± 1	37 ± 1	2.0 ± 0.02	1.2 ± 0.05	**567 ± 7**
	3 m	0.40 ± 0.01	6.8 ± 0.4	69 ± 5	22 ± 1	329 ± 22	38 ± 2	1.8 ± 0.1	44 ± 3	52 ± 4	2.7 ± 0.2	2.5 ± 0.2	**830 ± 65**
P5	0	0.01 ± 0.001	0.01 ± 0.001	1.5 ± 0.1	0.20 ± 0.02	10 ± 1	1.1 ± 0.1	0.01 ± 0.001	1.1 ± 0.02	3.0 ± 0.1	0.05 ± 0.003	0.05 ± 0.001	3.0 ± 0.5
	6 h	0.10 ± 0.02	0.50 ± 0.10	15.0 ± 0.7	7.3 ± 0.2	109 ± 12	9.1 ± 0.8	0.70 ± 0.05	14 ± 1	128 ± 7	0.70 ± 0.02	0.60 ± 0.02	57 ± 2
	3 m	0.20 ± 0.02	1.0 ± 0.1	24 ± 2	14 ± 1	124 ± 10	10 ± 1	1.3 ± 0.1	23 ± 1	220 ± 19	1.1 ± 0.05	1.8 ± 0.1	92 ± 8
P6	0	0.02 ± 0.001	0.10 ± 0.01	5 ± 0.3	0.20 ± 0.02	13 ± 1	1.2 ± 0.1	0.10 ± 0.01	2.0 ± 0.2	4.0 ± 0.2	0.50 ± 0.03	0.05 ± 0.001	9 ± 1
	6 h	0.60 ± 0.01	4.4 ± 0.6	106 ± 9	22 ± 1	612 ± 47	37 ± 2	2.7 ± 0.1	69 ± 7	25 ± 1	4.2 ± 0.6	1.7 ± 0.01	**478 ± 21**
	3 m	0.70 ± 0.03	11 ± 0.5	204 ± 15	28 ± 2	887 ± 66	58 ± 5	4.1 ± 0.4	102 ± 8	41 ± 3	8.1 ± 0.4	3.0 ± 0.2	**1050 ± 90**
P7	0	0.10 ± 0.01	0.30 ± 0.02	**5.7 ± 0.4**	3.0 ± 0.1	14 ± 1	5.6 ± 0.5	0.10 ± 0.01	3.0 ± 0.2	6.0 ± 0.2	0.30 ± 0.02	0.2 ± 0.02	13 ± 1
	6 h	0.50 ± 0.10	3.0 ± 0.2	**171 ± 6**	32 ± 2	**728 ± 55**	50 ± 4	2.7 ± 0.1	63 ± 3	45 ± 2	3.7 ± 0.2	2.7 ± 0.1	223 ± 4
	3 m	0.60 ± 0.1	4.8 ± 0.3	**333 ± 24**	75 ± 6	**1170 ± 100**	77 ± 6	5.6 ± 0.4	96 ± 7	72 ± 5	4.9 ± 0.3	3.8 ± 0.2	280 ± 27
B5	0	0.02 ± 0.001	0.01 ± 0.001	0.30 ± 0.03	**24 ± 2**	11 ± 1	0.02 ± 0.001	0.01 ± 0.001	0.05 ± 0.002	**48 ± 2**	0.50 ± 0.04	0.05 ± 0.001	39 ± 3
	6 h	0.30 ± 0.03	0.10 ± 0.001	7.8 ± 0.6	**972 ± 83**	155 ± 6	0.50 ± 0.02	0.10 ± 0.02	1.0 ± 0.03	**8550 ± 920**	13 ± 0.4	0.60 ± 0.10	**1820 ± 40**
	3 m	0.40 ± 0.01	0.11 ± 0.01	11 ± 1	**1130 ± 110**	189 ± 14	0.60 ± 0.03	0.11 ± 0.01	1.3 ± 0.1	**9540 ± 810**	14 ± 1	0.70 ± 0.05	**2010 ± 180**
P8	0	0.30 ± 0.02	0.20 ± 0.02	0.7 ± 0.04	3.0 ± 0.2	**112 ± 10**	**28 ± 2**	0.30 ± 0.03	0.80 ± 0.03	11 ± 0.3	22 ± 1	0.40 ± 0.02	77 ± 4
	6 h	**8.5 ± 0.3**	2.4 ± 0.4	13.8 ± 0.6	82 ± 3	**6330 ± 980**	**1060 ± 90**	3.8 ± 0.2	33 ± 1	249 ± 9	**343 ± 10**	10 ± 0.4	**20,600 ± 1500**
	3 m	**9.2 ± 0.6**	2.9 ± 0.2	16.1 ± 1.5	99 ± 8	**7020 ± 520**	**1130 ± 100**	5.1 ± 0.3	44 ± 3	297 ± 22	**411 ± 25**	13 ± 0.5	**23,600 ± 2000**

^a^ m = month.

## Data Availability

Not applicable.

## Supplementary Materials

The following supporting information can be downloaded at https://www.mdpi.com/article/10.3390/polym15061523/s1, Table S1: List of analyzed plastic samples; Table S2: Carbon and hydrogen isotope composition of IAEA-CH-7 at varying sample mass; Figure S1: Time-dependent variations of δ^13^D; Table S3: Removal of metals from plastic material.
