# Supplementary material for: Mass Spectrometry Insight for Assessing the Destiny of Plastics in Seawater
Source: Polymers (Basel). 2023 Mar 19;15(6):1523. doi: 10.3390/polym15061523 (PMC10052999; doi:10.3390/polym15061523)
Supplement: Supplementary file 1 [file polymers-15-01523-s001.zip › polymers-2271831-supplementary.pdf]

Supplementary Materials

Mass Spectrometry Insight for Assessing the Destiny of Plastics in Seawater

Olga V. Kuznetsova [1], Sergey N. Shtykov [2] and Andrei R. Timerbaev [2,3,*]

[1] Vernadsky Institute of Geochemistry and Analytical Chemistry, 119991 Moscow, Russia
[2] Institute of Chemistry, Saratov State University, 410012 Saratov, Russia
[3] Institute of Inorganic Chemistry, University of Vienna, 1090 Vienna, Austria
* Correspondence: andrei.timerbaev@univie.ac.at

Contents

Table S1. List of analyzed plastic samples
Table S2. Carbon and hydrogen isotope composition of IAEA-CH-7 at varying sample mass
Figure S1. Time-dependent variations of δ^{13}D
Table S3. Removal of metals from plastic material

Table S1. List of analyzed plastic samples.

Code	Sample	Dimensions (mm)	Photo	Plastic type [a]
B1	Biodegradable knife	160 × 14 × 1	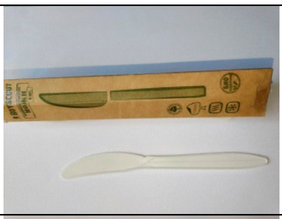	Starch PP
B2	Bio make-up sponge	55 × 37	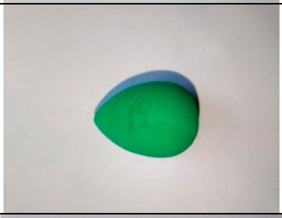	Starch
B3	Biodegradable waste bags	125 ×60	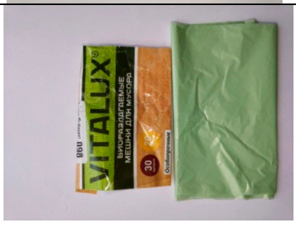	LD PE
B4	Biodegradable seedling bags	145 × 60	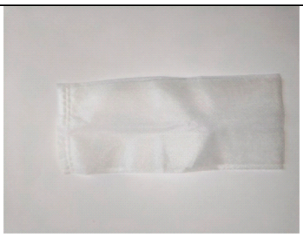	S-PP

N1	Part of the food package	56 × 31	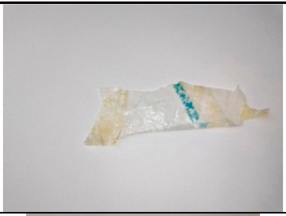	PP
N2	Part of the food package	60 × 22	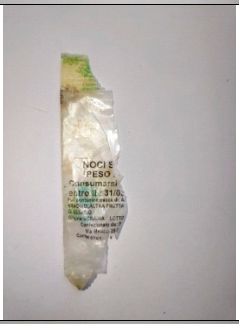	PP
N3	Part of the food package	70 × 91	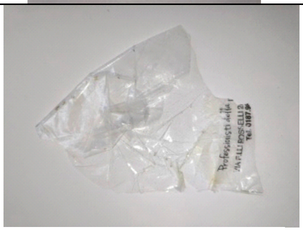	PP
N4	Pad	55 × 55	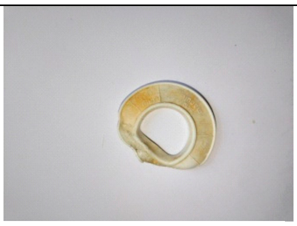	PVC
N5	Part of the food package	64 × 78	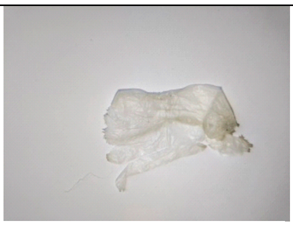	PE
N6	Part of the food package	116 × 76	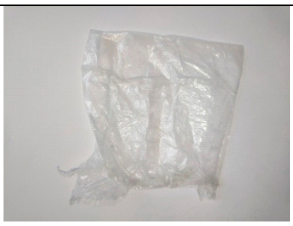	PE
N7	A piece of Teflon mat	8 × 14	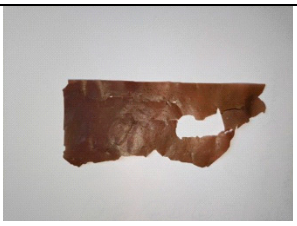	PTFE

N8	A piece of dispos-able cup	62 × 22	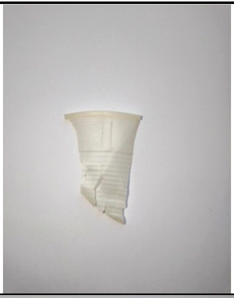	PP
N9	Medicine blister	96 × 39	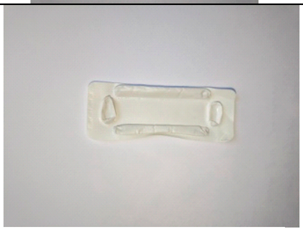	PP
N10	Part of the food package	53 × 60	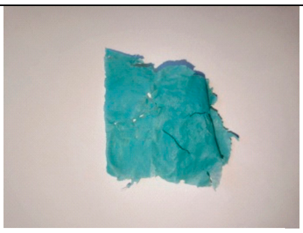	PE
N11	Packaging materi-al	112 × 80 × 0.3	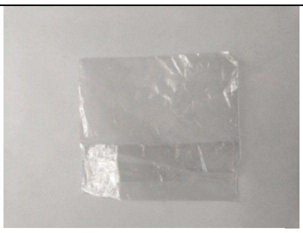	PP
P1	Packaging from capsules for the elemental analyz-er	80 × 30	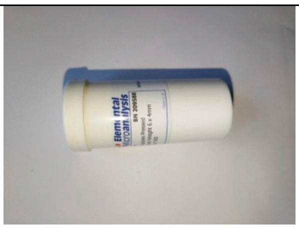	PE
P2	Test tube	119 × 16	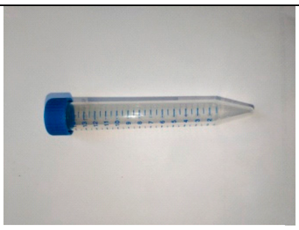	PP
P3	Test Eppendorf tube	17 × 55	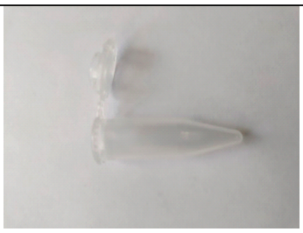	Virgin PP

P4	Disposable fork	162 × 23	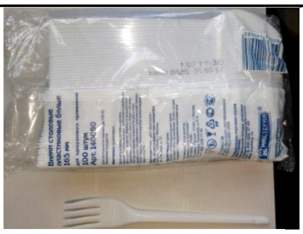	PS
P5	Disposable spoon	164 × 37	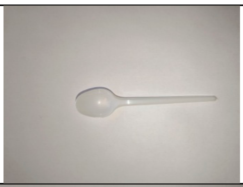	PS
P6	Disposable cup	95 × 65	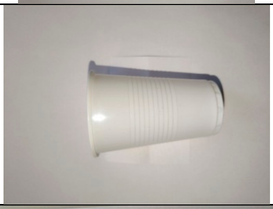	PP
P7	Packaging materi-al	232 × 147	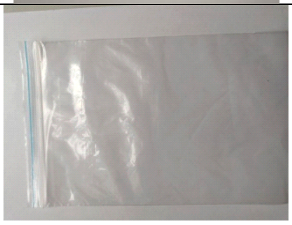	LD PE
B5	Biodegradable ball airsoft	5 × 5	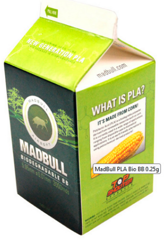	PLA
P8	Ball airsoft	5 × 5	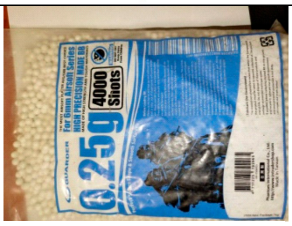	ABS
B6	Bio food contain-er	190 × 145 × 70	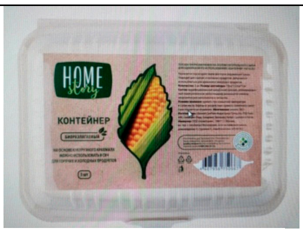	Starch PP
P9	Teflon mat	400 × 300	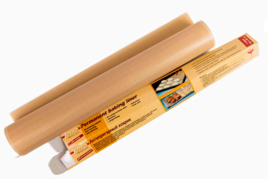	PTFE

[a] PE = polyethylene; PP = polypropylene; LD PE = low density PE; S-PP = spunbond PP; PVC = polyvinylchloride; PTFE = polytetrafluoroethylene; virgin PP = PP free of bio-

cides, plasticizers and latex; PS = polystyrene; PLA = polylactide; ABS = acrylonitrile butadiene styrene; starch PP = modified corn starch agglomerated with PP, monoglyceride, ethylene vinyl acetate; starch = tapioca starch, corn starch, potato starch.

Table S2. Carbon and hydrogen isotope composition of IAEA-CH-7 at varying sample mass.

Mass (mg)	$\delta^{13}C$	δD
0.41	–31.97	–100.24
0.98	–31.72	–98.18
1.37	–31.81	–98.23
1.86	–31.77	–99.31
2.34	–31.55	–100.16
2.66	–31.46	–98.84
3.05	–31.93	–100.34
3.49	–31.74	–98.89
4.12	–31.99	–97.88
4.68	–31.43	–99.16
5.15	–32.00	–100.12

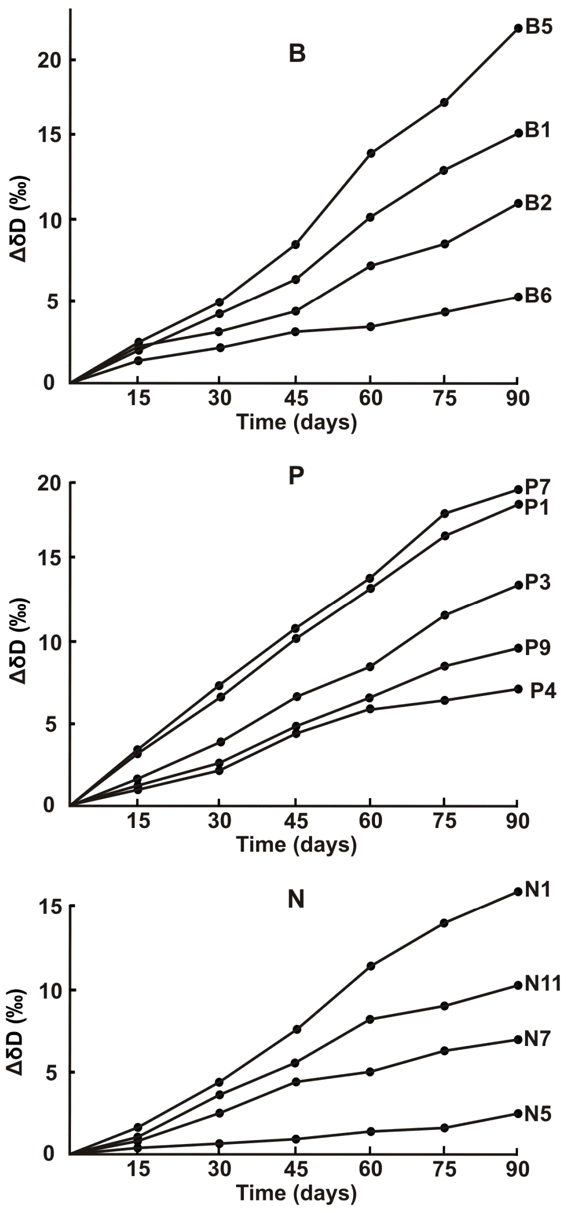

Figure S1. Time-dependent variations of $\delta^{13}D$. Indices B, P and N correspond to the respective polymer classes.

Table S3. Removal of metals from plastic material.

Metal	Sample	Concentration in Plastic Leachates (µg·g^{-1})			
		**5%**	**10%**	**20%**	**30%**
**Cd**	N6	0.007 ± 0.001	0.009 ± 0.001	0.021 ± 0.005	0.020 ± 0.001
	N7	0.03 ± 0.003	0.04 ± 0.003	0.010 ± 0.002	0.11 ± 0.005
Co	N6	0.20 ± 0.02	0.50 ±0 .01	0.60 ± 0.02	1.2 ± 0.07
	N7	0.50 ± 0.05	0.70 ± 0.04	0.90 ± 0.04	1.2 ± 0.1
Cu	N6	3.9 ± 0.3	4.0 ± 0.2	4.9 ± 0.3	5.6 ± 0.4
	N7	4.1 ± 0.3	5.5 ± 0.3	6.6 ± 0.5	7.8 ± 0.4
Cr	N6	4.3 ± 0.3	4.4 ± 0.4	4.8 ± 0.3	5.0 ± 0.4
	N7	4.3 ± 0.4	4.5 ± 0.5	4.7 ± 0.2	5.2 ± 0.3
Fe	N6	85 ± 7	87 ± 7	92 ± 8	95 ± 7
	N7	84 ± 8	89 ± 6	95 ± 6	99 ± 8
Mn	N6	5.4 ± 0.6	5.3 ± 0.4	5.8 ± 0.5	6.3 ± 0.6
	N7	46 ± 4	51 ± 5	55 ± 9	57 ± 6
Mo	N6	0.11 ± 0.01	0.15 ± 0.01	0.18 ± 0.01	0.22 ± 0.02
	N7	1.2 ± 0.1	1.5 ± 0.1	1.6 ± 0.2	1.7 ± 0.1
Ni	N6	5.4 ± 0.4	5.8 ± 0.3	6.0 ± 0.5	6.0 ± 0.5
	N7	4.0 ± 0.2	4.6 ± 0.4	4.9±0.4	5.1 ± 0.5
Pb	N6	0.43 ± 0.03	0.46 ± 0.04	0.5±0.05	0.51 ± 0.04
	N7	5.1 ± 0.4	5.4 ± 0.6	5.6±0.4	5.8 ± 0.5
Ti	N6	0.49 ± 0.03	0.54±0.02	0.57±0.03	0.62 ± 0.05
	N7	2.5 ± 0.2	3.0±0.3	3.1±0.3	3.3 ± 0.2
V	N6	0.16 ± 0.01	0.18±0.01	0.2±0.01	0.22 ± 0.02
	N7	0.45 ± 0.05	0.47±0.03	0.5±0.04	0.53 ± 0.04
Zn	N6	14.3 ± 1.2	14.5±1.2	14.8±1.3	15.0 ± 1.0
	N7	15.1 ± 1.0	15.5±1.2	15.8±1.1	16.1 ± 1.3
